# A Recipe for Success

**DOI:** 10.1111/imcb.12786

**Published:** 2024-06-02

**Authors:** Maike de la Roche

**Affiliations:** Cancer Research UK Cambridge Research Institute, Li Ka Shing Centre Robinson Way, Cambridge CB2 0RE, UK

**Keywords:** Translational immunology, career, life-work balance

It’s hard to encapsulate what success means in science. Is it impactful science? A tenured faculty position? Or perhaps the legacy of scientists you have mentored?

For me, success is a combination of the above, but in reality it must include a healthy work life balance. As for a Recipe for Success, I can only speak to my own experiences and throughout my career there have been some constants that have been essential. My key ingredients are as follows:

## Engaging science

only you can decide what type of science is engaging to you and by definition this is Great science. If you read about something and say to yourself “that is really cool!”, this is a good indicator that this will be engaging science to you. But also keep in mind that you will spend years at a time focussing on a given problem. When picking your research area two good litmus tests are (1) you immediately see the fundamental importance of the research question and (2) your enthusiasm for the topic means that you can make it exciting to others who do not have a background in science. One thing to note, one of the biggest challenge in science is to know when to persevere and finish a project and when to admit when a line of research is not working and further attempts are futile. While the determination to complete a project ultimately separates the good from the excellent scientists, knowing when to stop also requires oversight and courage. A tricky balance that is not easy to get right from the very beginning. Choose supervisors and mentors, who are able to guide you.

## Tenacity

Famously, Louis Pasteur once said: “Let me tell you the secret that led me to my goal. My sole strength is in my tenacity.”. Persistence and determination are an accompaniment to both lab bench level research (see above) and career progression. Career progression is a marathon and does not happen overnight. It is a slow and sometimes disheartening process, but you have to stick with it.

## Supportive Mentors

**Figure 1:**
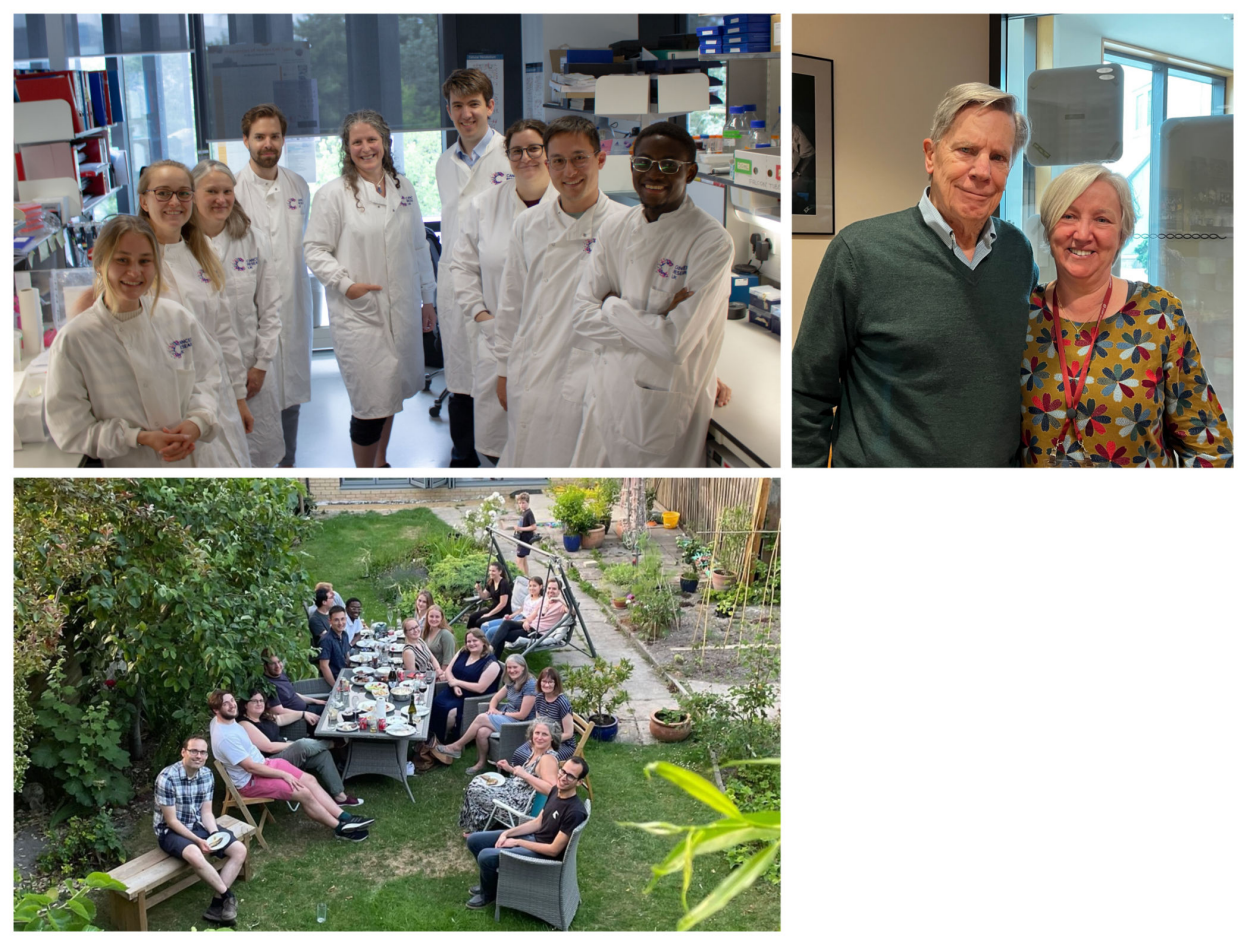
One of the best things in science. *top left:* the lab at the Cancer Research UK Cambridge Institute. *top right:* my supervisors and mentors Prof Gillian Griffiths (during my postdoctoral studies) and Prof Doug Fearon (during my PhD), whose world leading science and support I was fortunate to experience throughout my career in science. *bottom left:* one of our low-key lab gatherings.

On top of being in the right place at the right time, having good mentors is the most important thing to a scientist at any level for career progression. Good mentors will be hypercritical when reviewing your papers and grant applications, prepare you for interviews, act as an agony aunt/uncle and unlock doors, some that you weren’t even aware of. During my career I have been beyond fortunate to having the most wonderful and amazing mentors (see Figure 1). They supported me when I had my children during my PhD and early postdoctoral years; they were there for me when I struggled with debilitating illness, and they have been instrumental as idea boards, proofing papers or for simple practical advice. Identify mentors early in your career and keep them close. While my PhD and postdoc supervisors have been incredible mentors, some supervisors operate in a different manner and you may find yourself in need of a more engaged mentor. There are mechanisms in place to help you out and I suggest engaging with mentorship programmes available through immunology societies (e.g. British Society of Immunology^a^, Australian and New Zealand Society for Immunology^b^, American Association of Immunologists^c^, …) or maybe even your own institution.

## Rich research environment

Publications in impactful immunology journals now require a wealth of resources ranging from expensive *in vivo* facilities, high-dimensional flow cytometry, single-cell omics and advanced bioinformatics. Choose your research environment with this in mind. If this is not possible, plan ahead, explore multiple funding options in parallel and choose your collaborators well. However, a rich research environment is not all about resources, it is also about people. Diversity in interdisciplinary expertise and cultural backgrounds is a catalyst of creativity and innovation. Play an active part: scientists who can effectively work as part of a team and collaborate well will be most successful. Be generous with your expertise, reagents and kindness and also willing to receive help, advice and critic from others.

## Colleagues

No person is an island. Seek out colleagues and establish ties. Discuss challenges (personal and academic) and compare notes. Commiserate when things go pear-shaped and celebrate each other’s successes – these sorts of activities flatten the lows and magnify the highs.

## Work–Life balance

Cultivating your interests beyond your research career is essential and will bring ‘true’ perspective to the pressures and disappointments that come with a career in science.

The Covid pandemic has made us all re-evaluate our work-life balances. It is now more important than ever to engage with activities outside of work, in particular, where you interact directly with other people. Covid has also forced us to road-test alternative working arrangements (e.g. part-working from home) that you now can evaluate to ease pressures that occur when building your research career and family life at the same time. Make use of them.

Careers in science have evolved. A career in academic science has become more challenging than ever – there is a paucity of funding opportunities across the board and the rising numbers of postgraduates continues to outpace the number of tenured faculty-level positions. On the other hand, professional science careers, for instance working in Pharma, have been on the rise, likely due to the increase in attractive jobs with competitive salaries, benefits, and regulated working hours. While a lot of what I have mentioned is geared towards academic research, the principles hold true for professional science – engaging science, tenacity, mentors, colleagues and work-life balance – essential ingredients in the Recipe for Success.

The immunology community needs you.^1^ We need your science and need to hear your opinions and ideas of how to make our research community fairer, more diverse and sustainable for the future!

(a) https://www.immunology.org/careers/bsi-careers-support/bsi-mentoring-scheme
(b) https://www.immunology.org.au/asi-programs-and-opportunities/mentor-mentee-program
(c) https://www.aai.org/Careers
